# Orbital Complications of Extensive Allergic Fungal Rhinosinusitis: A Case Report

**DOI:** 10.7759/cureus.39555

**Published:** 2023-05-27

**Authors:** Salmah M Alharbi, Ali Alzarei, Talat Ardi, Norah F Saleh, Mohammed Al Hamoud, Khalid Al Malwi, Mohammed Asiri, Sharefah Ahmed

**Affiliations:** 1 Otorhinolaryngology-Head and Neck Surgery Department, Aseer Central Hospital, Abha, SAU; 2 Family Medicine Department, Alqabel Primary Health Care Center, Ministry of Health, Abha, SAU; 3 Faculty of Medicine, King Khalid University, Abha, SAU

**Keywords:** proptosis, orbital, ophthalmic, fungal sinusitis, allergy

## Abstract

Allergic fungal rhinosinusitis is an immunoglobulin E-mediated disease caused by fungal antigens. Orbital complications due to bone erosion by the expanding, mucin-filled sinuses are considered uncommon, but they require immediate intervention. We report a successful management of a complicated case of allergic fungal rhinosinusitis in a 16-year-old female complaining of progressive nasal obstruction for four months, who only sought medical advice after developing proptosis and visual affection. The patient underwent surgical debridement and corticosteroid therapy followed by dramatic improvement of proptosis and vision. The differential diagnosis of proptosis with sinusitis must include allergic fungal rhinosinusitis.

## Introduction

Allergic fungal rhinosinusitis (AFRS) is a subtype of chronic rhinosinusitis with nasal polyps. Patients usually complain of purulent rhinorrhea and progressive nasal obstruction [1]. Bent-Kuhn criteria, commonly used for the diagnosis, consist of type-1 hypersensitivity to fungal elements, nasal polyps, characteristic computed tomography (CT) findings, eosinophilic mucus without fungal invasion, and positive fungal stain [2]. Surgical debridement is the mainstay of treatment [3].

In severe cases of AFRS, sinuses become large and filled with allergic mucin. The expanding sinuses can impinge upon adjacent spaces including the orbit. The most common symptom of orbital involvement is proptosis in addition to ophthalmoplegia [4]. Visual affection is suggested to be secondary to optic neuritis. Central retinal artery occlusion resulting from elevated intraorbital pressure is another mechanism [4]. Once there is visual affection, it should be managed urgently. A delay may lead to irreversible blindness [5]. Here, we report the successful management of AFRS with orbital complications in a 16-year-old female whose main complaint at presentation was proptosis.

## Case presentation

**Figure 1:**
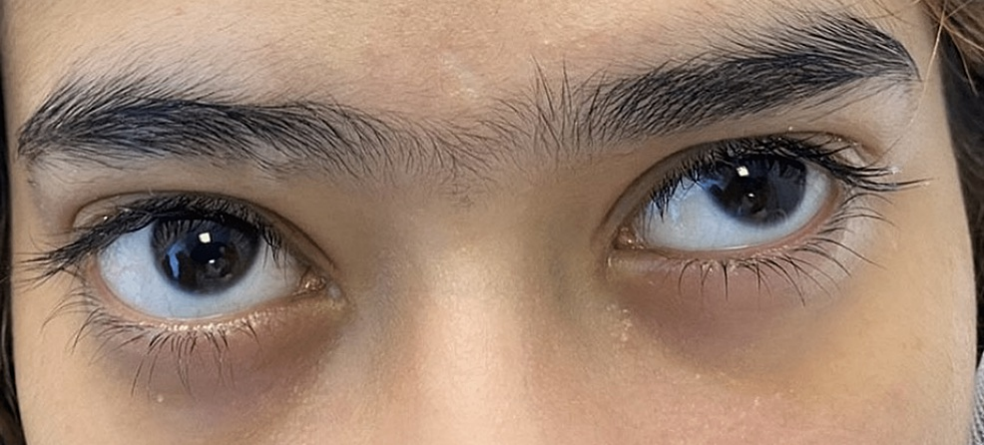
Proptosis of the Right Eye

A 16-year-old Saudi female was referred to our institute with a diminution of vision in the right eye for three days reaching almost no light perception one day before her presentation. She also complained of mild pain in the right eye, right periorbital swelling, and nasal discharge. The condition started four months earlier with right-sided facial pain as well as right-sided nasal obstruction, discharge, and hyposmia. One month before the presentation, the patient started to have blurring of vision. There was no history of fever, facial numbness, weight loss, night sweating, loss of consciousness, bronchial asthma, diabetes mellitus, chronic illnesses, medication intake, or smoking. She was not in contact with sick persons, and there was no history of travel. On examination, the patient was fully conscious and vitally stable. She had mild proptosis of the right eye with periorbital swelling (Figure 1).

**Figure 2:**
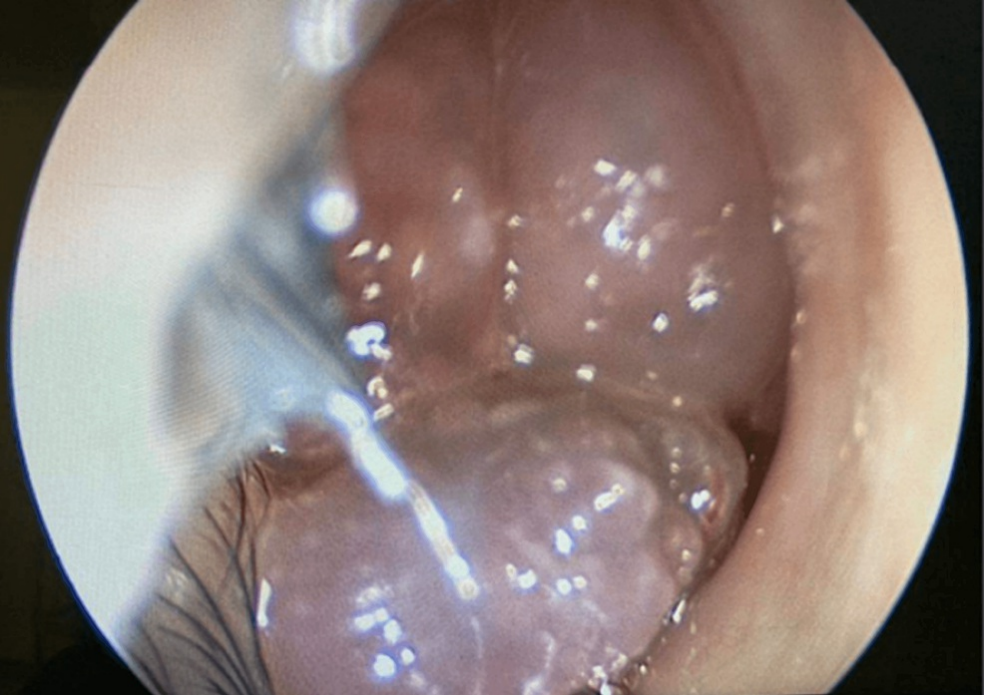
Anterior Rhinoscopy Showing Grade Four, Right-Sided Nasal Polyps With Nasal Septum Deviation to the Left

The extraocular muscles were completely mobile with slight pain. Examination of the other ear, nose, and throat together with systemic review was unremarkable. Anterior rhinoscopy showed grade four right-sided nasal polyps with nasal septum deviation to the left (Figure 2).

**Figure 3:**
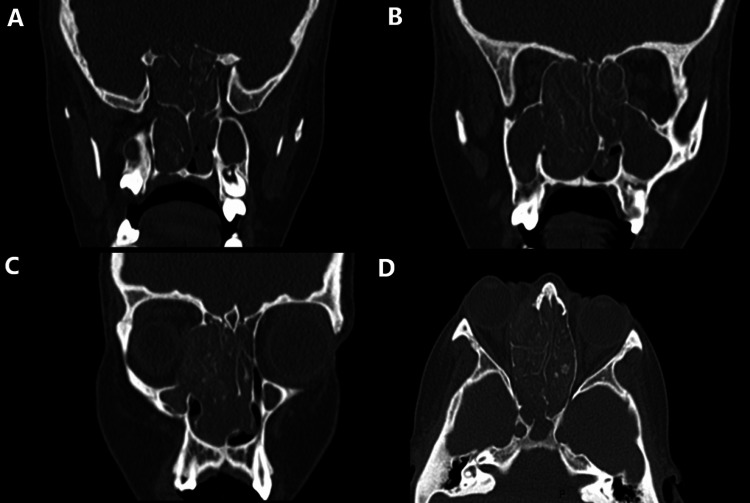
Computed Tomography With Coronal Section of Paranasal Sinuses Showing Right-Sided (A) Intracranial Extension From the Sphenoid Sinus, (B) Fully Opacified Sinuses With Fungi, (C) Intraorbital Extension, and (D) Axial Section Showing Full Opacification With Heterogeneous Densities Indicating Fungal Infection

The patient was admitted, routine laboratory investigations were ordered, and urgent ophthalmological consultation was requested. The ophthalmologist confirmed normal extraocular muscle movement but with mild pain. The vision in the left eye was normal (6/6), while the right eye’s vision was fingers counting. The intraocular pressure was 19 mmHg and 17 mmHg in the right and left eyes, respectively. The right optic disc was hyperemic and suggestive of ischemic compressive optic neuropathy. CT was done (Figure 3) and the patient was diagnosed with AFRS with orbital involvement and urgent intervention was recommended.

**Figure 4:**
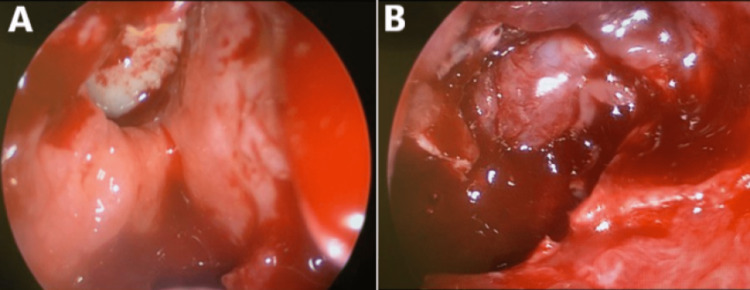
Intra-operative Endoscopic Picture Showing (A) Right Sphenoid Sinus and (B) Dehiscence of the Roof of Sphenoid Sinus and Exposure of the Optic Nerve

Once admitted, the patient was started on intravenous dexamethasone (8 mg) three times daily. She was scheduled for emergency functional endoscopic sinus surgery, and informed consent was obtained from the patient after fully explaining to the patient and her relatives that vision loss may be irreversible. Intra-op finding of completely exposed optic nerve on the right eye with intra-carinal extension through the sphenoid sinus, pulsation of dura was noticed (Figure 4).

**Figure 5:**
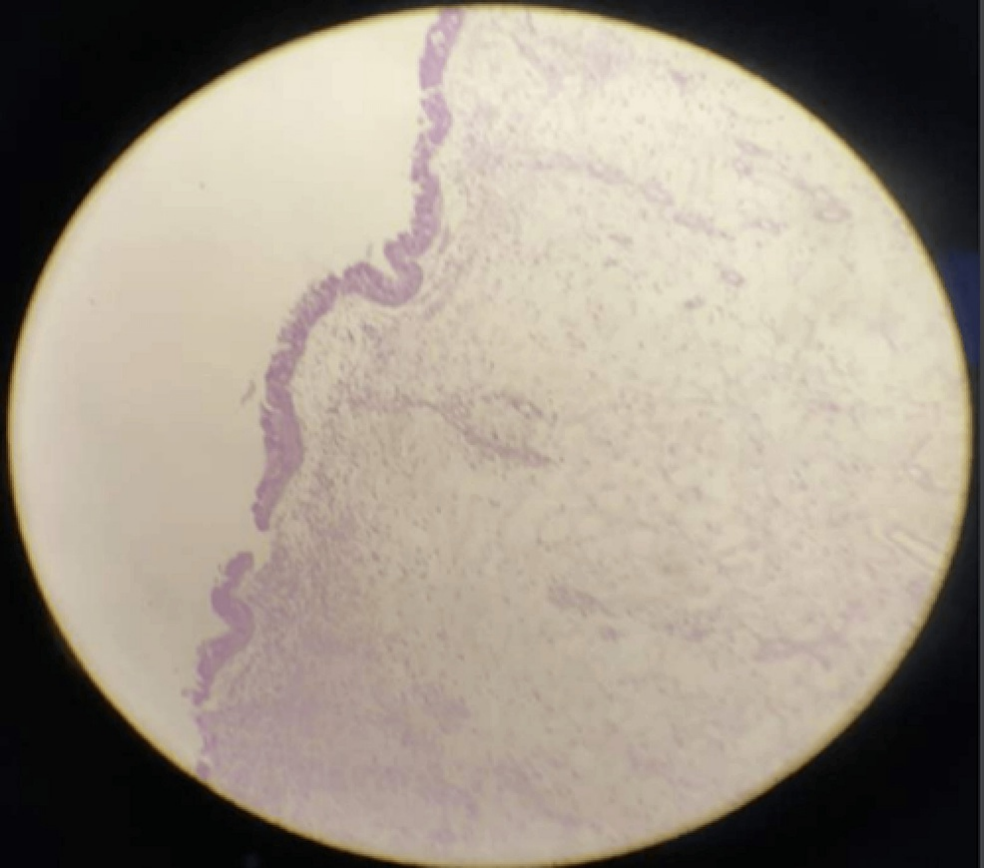
Histopathology Consistent With Inflammatory Polyps Where no Fungal Hyphae Were Seen

Histopathology was consistent with inflammatory polyps, but no fungal hyphae were seen (Figure 5).

**Figure 6:**
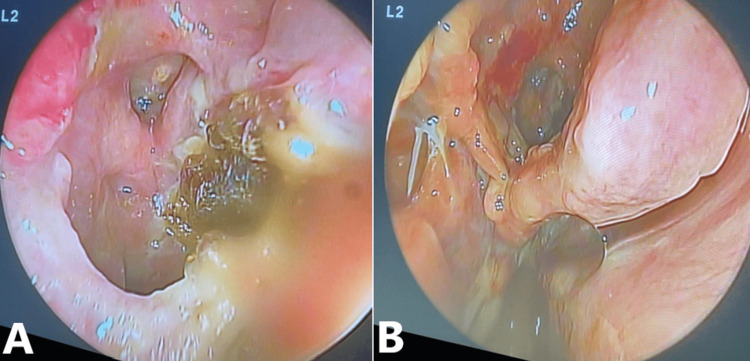
Postoperative Right-Sided Nasal Endoscopy Images Showing (A) Right Sphenoid Sinus and (B) Dehiscence of the Roof of Sphenoid Sinus and Exposure of the Optic Nerve

Postoperatively, the patient was started on oral corticosteroids (1 mg/kg) once daily, Augmentin (625 mg) three times daily, intravenous paracetamol (1 g) every six hours, intranasal Pulmicort irrigation two times daily, and Xylomet nasal drops three times daily. Postoperatively, the pain, visual acuity, and pupil reaction improved. Besides, the right optic disc looked healthy, and extraocular muscle movement was normal. However, color vision in the right eye was still affected. The patient was discharged home after three days on oral corticosteroid (1 mg/kg) once daily for one week after which the dose would be tapered, Augmentin (625 mg) three times daily for one week, oral paracetamol (1 g) every six hours for one week, in addition to intranasal Pulmicort irrigation twice daily, and Xylomet nasal drops three times daily for five days. The patient looked normal on follow-up two weeks postoperatively (Figure 6).

A postoperative evaluation revealed that the visual acuity and intraocular pressure of the right and left eyes were 20/150 versus 20/20 and 19 mm Hg versus 17 mm Hg, respectively. Color vision was reduced in the right eye, but the extraocular muscles and the optic discs were normal in both eyes.

## Discussion

This is a case of a 16-year-old female suffering from orbital complications following AFRS. The presence of unilateral proptosis and visual affection preceded by a history of nasal symptoms and nasal polyposis raised suspicion for AFRS. The age and gender of the patient were consistent with the literature [6]. This prompted the confirmation of orbital involvement with CT, the best investigation to start with when AFRS is suspected.

The patient was urgently started on intravenous corticosteroids. Earlier studies recommend oral corticosteroids as a preoperative short course in AFRS. However, when vision is affected, intravenous steroids are recommended [7].

In AFRS, hematoxylin and eosin staining usually shows eosinophilic mucin and mucosal infiltration with inflammatory cells as demonstrated in our case. Fungal hyphae are seen in most cases as a negative image [8], but they may be absent as reported in about one-third of cases assessed by Salamah et al. [3].

Emergency functional endoscopic sinus surgery was performed on our patient as recommended whenever vision is threatened. The rapid improvement in our patient’s condition is consistent with the literature, which correlates bad prognosis of visual loss with delay in seeking help. The surgery lacks significant benefit in patients with a marked delay of more than a month after visual loss and cases with complete loss [5].

Postoperative oral steroid therapy and nasal irrigation improve symptoms and prevent early recurrences [6]. A recent clinical trial reported the comparable efficacy of Itraconazole and methylprednisolone in preventing disease recurrence of AFRS when steroids are contraindicated [9]. Recent evidence is increasing to support the benefits of adding dupilumab to daily care for patients with severe, chronic sinusitis and nasal polyps with multiple recurrences [10].

## Conclusions

The differential diagnosis of patients with sinusitis and proptosis must include AFRS. Otolaryngologists and ophthalmologists should be familiar with the possible orbital complications of AFRS. Early suspicion, besides medical and surgical intervention, can salvage the patient’s vision.

## References

[1] AlAhmari AA (2021). Allergic fungal rhinosinusitis in Saudi Arabia: a review of recent literature. Cureus.

[2] Bent JP, 3rd 3rd, Kuhn FA (1994). Diagnosis of allergic fungal sinusitis. Otolaryngol Head Neck Surg.

[3] Salamah MA, Alsarraj M, Alsolami N, Hanbazazah K, Alharbi AM, Khalifah W Sr (2020). Clinical, radiological, and histopathological patterns of allergic fungal sinusitis: a single-center retrospective study. Cureus.

[4] Al Dousary S (2011). Ophthalmic manifestations of allergic fungal sinusitis. Saudi J Otorhinolaryngol Head Neck Surg.

[5] Thakar A, Lal P, Dhiwakar M, Bahadur S (2011). Optic nerve compression in allergic fungal sinusitis. J Laryngol Otol.

[6] Alghonaim Y, Alfayez A, Alhedaithy R, Alsheikh A, Almalki M (2020). Recurrence pattern and complication rate of allergic fungal sinusitis: a 10-year tertiary center experience. Int J Otolaryngol.

[7] Vashishth A (2015). Extensive allergic fungal rhinosinusitis: ophthalmic and skull base complications. Indian J Otolaryngol Head Neck Surg.

[8] Chakrabarti A, Kaur H (2016). Allergic aspergillus rhinosinusitis. J Fungi (Basel).

[9] Salil A, Joy N, Faizal B (2023). A prospective study comparing itraconazole alone versus systemic steroids alone as adjuncts to topical steroids in the post-operative management of allergic fungal rhinosinusitis. Clin Otolaryngol.

[10] Bachert C, Han JK, Desrosiers M (2019). Efficacy and safety of dupilumab in patients with severe chronic rhinosinusitis with nasal polyps (LIBERTY NP SINUS-24 and LIBERTY NP SINUS- 52): results from two multicentre, randomised, double-blind, placebo-controlled, parallel-group phase 3 trials. Lancet.

